# A phase II trial of ramucirumab and docetaxel as second-line treatment for patients with advanced gastric cancer (HGCSG 1903)

**DOI:** 10.1007/s10120-026-01751-w

**Published:** 2026-05-04

**Authors:** Yasuyuki Kawamoto, Kentaro Sawada, Kazuaki Harada, Iori Motoo, Takayuki Ando, Susumu Sogabe, Yoshimitsu Kobayashi, Masayoshi Dazai, Michio Nakamura, Kazuteru Hatanaka, Atsushi Ishiguro, Atsushi Sato, Shintaro Nakano, Yoshiaki Shindo, Ayumu Hosokawa, Ken Ito, Ayumu Yoshikawa, Akira Ueda, Kayoko Iuchi, Isao Yokota, Satoshi Yuki, Yoshito Komatsu

**Affiliations:** 1 Division of Cancer Center, Hokkaido University Hospital, Kita 14, Nishi 5, Kita-Ku, Sapporo, 060-8648 Japan; 2 Department of Medical Oncology, Kushiro Rosai Hospital, Kushiro, Japan; 3 Third Department of Internal Medicine, University of Toyama, Toyama, Japan; 4 Department of Medical Oncology, KKR Sapporo Medical Center, Sapporo, Japan; 5 Department of Gastroenterology, Sapporo Medical Center NTT EC, Sapporo, Japan; 6 Department of Gastroenterology, Sapporo City General Hospital, Sapporo, Japan; 7 Department of Gastroenterology, Hakodate Municipal Hospital, Hakodate, Japan; 8 Department of Medical Oncology, Teine Keijinkai Hospital, Sapporo, Japan; 9 Department of Medical Oncology, Hirosaki University Graduate School of Medicine, Hirosaki, Japan; 10 Department of Gastroenterology, Iwamizawa Municipal General Hospital, Iwamizawa, Japan; 11 Department of Gastroenterological Surgery, Nakadori General Hospital, Akita, Japan; 12 Department of Clinical Oncology, University of Miyazaki Hospital, Miyazaki, Japan; 13 Department of Gastroenterology, Tomakomai City Hospital, Tomakomai, Japan; 14 Department of Gastroenterology, Japanese Red Cross Kitami Hospital, Kitami, Japan; 15 Department of Gastroenterology, Toyama Red Cross Hospital, Toyama, Japan; 16 Data Center, Hokkaido Gastrointestinal Cancer Study Group, Sapporo, Japan; 17 Department of Biostatistics, Hokkaido University Graduate School of Medicine, Sapporo, Japan; 18 Department of Gastroenterology and Hepatology, Hokkaido University Hospital, Sapporo, Japan

**Keywords:** Advanced gastric cancer (AGC), Ramucirumab, Docetaxel, Second-line treatment

## Abstract

**Background:**

Ramucirumab has shown efficacy in combination with paclitaxel in the second-line treatment of advanced gastric cancer (AGC). The efficacy and safety regarding the combination therapy of ramucirumab and docetaxel have not been reported. This treatment could reduce the incidence of neuropathy and patients’ hospital visits.

**Methods:**

This was a multicenter, single-arm phase II trial. Patients with AGC who were refractory or intolerant to primary treatment were eligible. Patients received ramucirumab at a dose of 8 mg/kg on day1 and 15, and docetaxel at a dose of 60 mg/m^2^ on day1 of a 28-day cycle. The primary endpoint was overall response rate (ORR). The secondary endpoints were progression-free survival (PFS), overall survival (OS), relative dose intensity, and safety.

**Results:**

A final analysis of efficacy and safety was performed in 35 patients. ORR was 25.7% (90% confidence interval [CI] 14.1–40.6) and disease control rate was 74.3% (90%CI 59.4–85.9). Median PFS and OS were 3.1 months (95%CI 2.1–4.2) and 11.5 months (95%CI 9.2–13.9), respectively. Grade 3 or higher adverse events that occurred in more than 10% of patients were neutropenia, leucopenia, febrile neutropenia, hypertension, and anorexia. The study protocol was amended to allow primary granulocyte-colony stimulating factor (G-CSF) prophylaxis during study period. There was no one incident febrile neutropenia after the protocol amendment. Peripheral sensory neuropathy occurred in 65.7% of all grades, with no incidence of grade 3 or higher.

**Conclusions:**

The combination therapy of ramucirumab and docetaxel demonstrated some efficacy for AGC. Caution is required regarding the occurrence of febrile neutropenia. This treatment might be considered as an option for second-line treatment of patients with AGC.

## Introduction

Gastric cancer can be cured with standard treatments such as endoscopic resection or surgical resection if it is at a stage where resection is possible. However, advanced gastric cancer (AGC) that is unresectable or recurrent has a poor prognosis overall among solid tumors. Systemic chemotherapy is standard treatment for such stages.

Based on the results of multiple clinical trials conducted to date, the standard first-line treatment for AGC has become combination of fluoropyrimidine and platinum drugs plus immune checkpoint inhibitors in human epidermal growth factor receptor 2 (HER2) and Claudin 18.2 (CLDN18.2)-negative gastric cancer [1–3]. In cases of HER2-positive gastric cancer, trastuzumab is used in place of or in combination with immune checkpoint inhibitors [4, 5]. The efficacy of zolbetuximab has been reported in cases of CLDN18.2-positive gastric cancer [6, 7].

Ramucirumab is a fully human monoclonal IgG1 antibody that targets vascular endothelial growth factor receptor 2 (VEGFR2) [8]. Ramucirumab specifically binds to VEGFR2 and inhibits angiogenesis by blocking the binding of ligands VEGF-A, VEGF-C, and VEGF-D. Ramucirumab promotes the inhibition and regression of tumor angiogenesis, normalizes residual blood vessels, and facilitates the delivery of anticancer drugs to tumors. As a result, it suppresses tumor cell proliferation and affects the environment surrounding tumor cells [9, 10]. Ramucirumab and paclitaxel combination therapy had become a standard regimen in the second-line treatment for AGC, since the RAINBOW trial showed that combination therapy increased overall survival compared with paclitaxel monotherapy [11]. However, the incidence of neuropathy in patients receiving ramucirumab and paclitaxel combination therapy has been reported at 70.6% and 38.3% for Japanese and Western patients, respectively [12]. This toxicity can sometimes interfere with the continuation of treatment. Whereas docetaxel monotherapy used to be one of standard second-line treatment of AGC [13, 14]. In addition, the combination therapy of ramucirumab and docetaxel was reported to cause neuropathy at an incidence of 17.1–23% in patients with lung cancer [15, 16].

Based on these results, we hypothesized that the combination therapy of ramucirumab and docetaxel for patients with gastric cancer could be as effective as the ramucirumab and paclitaxel combination therapy and could reduce the incidence of neuropathy. In addition, in the RAINBOW trial, paclitaxel was administered weekly, whereas docetaxel only needs to be administered once every three to four weeks. Therefore, we thought that changing the combination with ramucirumab from paclitaxel to docetaxel would reduce patients’ hospital visits. We conducted this trial to examine the efficacy and safety of new combination therapy with ramucirumab and docetaxel in the second-line treatment for patients with AGC.

## Material and methods

### Study design and patients

HGCSG1903 was a single-arm, phase II trial carried out at 20 institutions in Japan. The key inclusion criteria were as follows: metastatic or advanced gastric cancer that is refractory or intolerant to initial chemotherapy; histologically or cytologically proven adenocarcinoma; aged 20 years or older; an Eastern Cooperative Oncology Group (ECOG) performance status of 0 or 1; a measurable lesion based on Response Evaluation Criteria in Solid Tumors (RECIST) version 1.1 within 28 days prior to enrollment; and a life expectancy of at least 12 weeks. The key exclusion criteria were as follows: any central nervous system metastasis; recent history of arterial or venous thromboembolism; recent history of severe gastrointestinal hemorrhage; and uncontrollable hypertension.

### Procedures

Patients received ramucirumab at a dose of 8 mg/kg on day1 and 15, and docetaxel at a dose of 60 mg/m^2^ on day1 of a 28-day cycle. Treatment was continued until disease progression or emergence of adverse events requiring discontinuation.

### Study endpoints and statistical methods

The primary endpoint was overall response rate (ORR) assessed by local investigator review according to RECIST version 1.1 criteria. The ORR to docetaxel monotherapy in second-line patients with AGC was reported to 10.7% [13]. In the RAINBOW trial, ORR of paclitaxel​ and ramucirumab combination therapy was reported to 27.9% [11]. Based on these results, we carried out the hypothesis test of binomial probability. The minimum sample size to achieve 80% power at a one-sided significance level of 5% was calculated to be 32, with a threshold of 10.7% for response rate and 27.9% for expectation. Some dropouts such as ineligibility were expected, and the target sample size was set at 35 patients.

The secondary endpoints were overall survival (OS), progression-free survival (PFS), relative dose intensity, and safety. OS and PFS were estimated using the Kaplan–Meier method. Adverse events were evaluated using the CTCAE v5.0 criteria. All statistical analyses were performed using IBM SPSS Statistic version 24.0 (IBM, Armonk, NY).

### Ethics and dissemination

The trial was conducted in accordance with the Declaration of Helsinki and the Clinical Trials Act in Japan. The trial received approval from the Hokkaido University Hospital Research Ethics Committee (020–002) and the Institutional Review Boards of each participating institution. All patients provided written informed consent prior to enrolling into the study. This trial was registered with the Japan Registry of Clinical Trials (jRCTs011200010).

## Results

### Patient characteristics and disposition

**Fig. 1:**
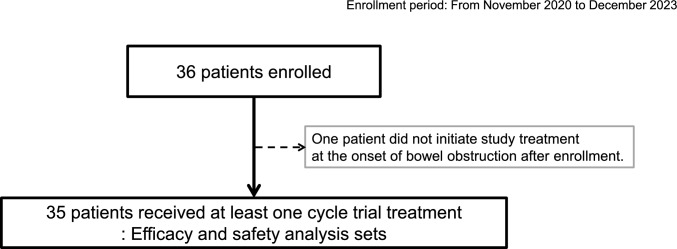
Consolidated Standards of Reporting Trials (CONSORT) flowchart. Thirty-six patients were enrolled. One patient did not initiate study treatment at the onset of bowel obstruction after enrollment. A final analysis of efficacy and safety was performed in 35 patients

Between November 2020 and December 2023, a total of 36 patients were enrolled. One patient did not initiate study treatment at the onset of bowel obstruction after enrollment. A final analysis of efficacy and safety was performed in 35 patients (Fig. 1).

Table 1 shows the baseline characteristics of the patients enrolled in the final analysis. The median age was 70 years (range, 36–82), with 7 female and 28 male patients. ECOG performance status was 0 for 16 patients and 1 for 19 patients. Thirteen patients were HER2-positive, and 12 of them had received first-line therapy including trastuzumab. Thirty-three (94%) patients had received a regimen including oxaliplatin as first-line therapy. Although ramucirumab was permitted as prior therapy, no patients had actually received it.

**Table 1:** Baseline characteristics

	*N* = 35	(%)
Age		
Median (range)	70	(36–82)
< 70	16	(46%)
≥ 70	19	(54%)
Sex		
Female	7	(20%)
Male	28	(80%)
ECOG performance status		
0	16	(46%)
1	19	(54%)
Disease status		
Advanced	28	(80%)
Recurrent	7	(20%)
Primary tumor site		
Gastroesophageal junction	6	(17%)
Stomach	29	(83%)
Histological subtype		
Intestinal	20	(57%)
Diffuse	13	(37%)
Unclassified	2	(6%)
HER2 status		
Negative	22	(63%)
Positive	13	(37%)
Metastatic site ¶		
Lymph node	26	(74%)
Peritoneum	16	(46%)
Liver	16	(46%)
Lung	9	(26%)
Others ⧻	7	(20%)
Prior gastrectomy		
Yes	10	(29%)
No	25	(71%)
First-line treatment		
FP + oxaliplatin + ICI	8	(23%)
FP + oxaliplatin + trastuzumab	12	(34%)
FP + oxaliplatin	13	(37%)
FP + CDDP	1	(3%)
FP	1	(3%)
Prior ramucirumab		
Yes	0	(0%)
No	35	(100%)

Data are presented as *N* (%) or median (range)

¶: data overlapping; ⧻: bone, 1; ovary, 2; adrenal, 1; ascites, 2; abdominal wall, 1

ECOG, eastern cooperative oncology group; HER2, human epidermal growth factor receptor 2; FP, fluoropyrimidine; ICI, immune checkpoint inhibitors; CDDP, cisplatin

### Study treatment delivery

The median number of doses of ramucirumab and docetaxel was 6 (range, 1–13) and 3 (range, 1–7), respectively. The median relative dose intensity of docetaxel and ramucirumab was 100% (range, 66.7–101.4%) for ramucirumab and 91.7% (range, 71.1–100.7%) for docetaxel. The reasons for discontinuation of study treatment were disease progression in 24 patients, adverse events in 10 patients, and one patient discontinued due to a fracture of the femur neck.

### Efficacy

**Fig. 2:**
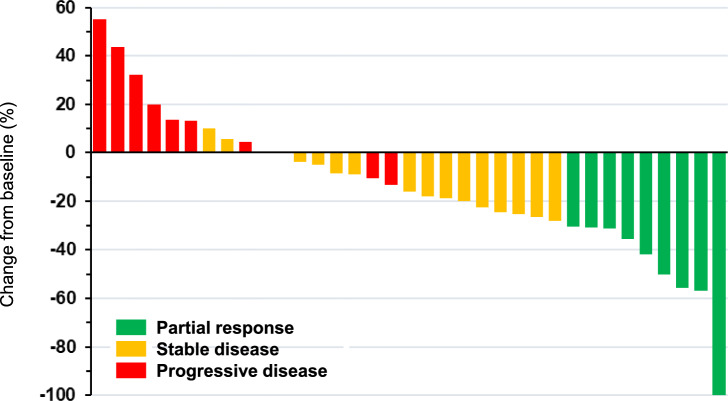
Waterfall plot of maximum tumor shrinkage. Overall response rate was 25.7% (90% confidence interval [CI] 14.1–40.6) and disease control rate was 74.3% (90%CI 59.4–85.9). Twenty-four of 35 assessed patients had some degree of tumor shrinkage

ORR was 25.7% (90% confidence interval [CI] 14.1–40.6) and disease control rate was 74.3% (90%CI 59.4–85.9) (Table 2). A waterfall plot of maximum tumor shrinkage showed that 24 of 35 assessed patients had some degree of tumor shrinkage (Fig. 2).

**Table 2:** Tumor response

	*N* = 35	
Partial response	9	
Stable disease	17	
Progressive disease	9	
Overall response rate	9	(25.7%; 90% CI 14.1–40.6)
Disease control rate	26	(74.3%; 90% CI 59.4–85.9)

Data are presented as *N* (%)

CI, confidence interval

**Fig. 3:**
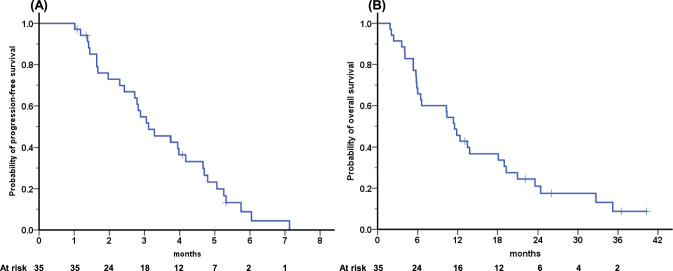
Progression-free survival (**A**) and overall survival (**B**). The median progression-free survival and overall survival were 3.1 months (95%CI 2.1–4.2) and 11.5 months (95%CI 9.2–13.9), respectively

The median PFS and OS were 3.1 months (95%CI 2.1–4.2) and 11.5 months (95%CI 9.2–13.9), respectively (Fig. 3).

Subgroup analyses for ORR, PFS, and OS were performed according to characteristics. Statistically significant differences were observed for PFS based on sex, and for OS based on sex, histological subtype, and HER2 status (Supplementary Table 1, Supplementary Fig. 1 and 2).

### Safety

Table 3 shows adverse events of any grade that occurred in at least 10% of the patients. Grade 3 or higher adverse events that occurred in more than 10% of patients were neutropenia (69%), leucopenia (57%), febrile neutropenia (17%), hypertension (14%), and anorexia (11%).

**Table 3:** Adverse events occurring in ≥ 10% of the patients, irrespective of causality

	All Grade		≥ Grade 3	
N = 35	n	%	n	%
Anemia	35	100	3	9
White blood cell decreased	25	71	20	57
Neutrophil count decreased	25	71	24	69
Platelet count decreased	22	63	0	0
Febrile neutropenia	6	17	6	17
Hypertension	32	91	5	14
Fatigue	28	80	2	6
Anorexia	27	77	4	11
Peripheral sensory neuropathy	23	66	0	0
Nausea	16	46	0	0
Alopecia	15	43	–	–
Proteinuria	13	37	0	0
Diarrhea	11	31	0	0
Vomiting	5	14	0	0
Epistaxis	4	11	0	0

At the time when 27 patients were enrolled in this trial, 6 patients experienced febrile neutropenia. Primary prophylactic administration of granulocyte-colony stimulating factor (G-CSF) was not permitted at the start of the trial. However, based on these findings during monitoring within the trial period, the study protocol was amended to allow primary prophylactic administration of G-CSF. Of the eight patients enrolled after the protocol amendment, seven patients received primary G-CSF prophylaxis. There was no one incident febrile neutropenia after the protocol amendment.

Peripheral sensory neuropathy occurred in 66% of all grades, with no incidence of grade 3 or higher.

No death and new safety signals with a causal relation to trial treatment were observed.

## Discussion

This trial is the first report evaluating the efficacy and safety of the combination therapy of ramucirumab and docetaxel in the second-line treatment for patients with gastric cancer. The primary endpoint of ORR met the pre-specified criteria, demonstrating the efficacy of this treatment. The PFS and OS results were comparable to previously reported data [11]. Unlike weekly paclitaxel administration, this treatment requires only biweekly visits, making it a good option for patients who would like to reduce the frequency of their visits.

The incidence of neutropenia and grade 1 or 2 peripheral sensory neuropathy was frequent. In the REVEL trial, which examined patients with non-small cell lung cancer that was refractory to platinum-based first-line therapy, the combination of ramucirumab and docetaxel showed high incidence rates of grade 3 or higher neutropenia (49%) and febrile neutropenia (16%) [15]. In particular, febrile neutropenia occurred at a high rate of 43.8% in East Asia. Therefore, the starting dose was modified, with 73% of patients receiving at 75 mg/m^2^ and 27% at 60 mg/m^2^[17]. In the JVCG trial conducted in Japan for patients with non-small cell lung cancer that was refractory to platinum-based first-line therapy, docetaxel 60 mg/m^2^ with ramucirumab was administered every 3 weeks. Febrile neutropenia occurred in 34.2% of patients, and G-CSF were used in 43.6% of patients [16]. In this trial, based on the results of the REVEL and JVCG trials, the treatment schedule consisted of docetaxel 60 mg/m^2^ administered every 4 weeks in combination with ramucirumab administered every 2 weeks. Febrile neutropenia was slightly frequent, and primary G-CSF prophylaxis may be helpful. Considering the incidence of febrile neutropenia, the balance between therapeutic efficacy and adverse events may require careful consideration. Additional visits associated with G-CSF prophylaxis could potentially undermine the benefit of this treatment in reducing overall visit frequency. The G-Lasta Subcutaneous Injection 3.6 mg BodyPod, which is an automated injection device, has been approved in Japan and may mitigate this weak point; however, this approach may not be feasible for all institutions. In actual clinicalç practice, it may be acceptable to start docetaxel at a reduced dose depending on the risk of the patient.

Peripheral sensory neuropathy of all grades was common, occurring in 66% of patients. However, no cases of grade 3 or higher neuropathy were observed. Of the 35 patients included in the safety analysis, 18 patients (51%) had peripheral sensory neuropathy at baseline, with 16 patients of grade 1 and two patients of grade 2. The situation has changed since the RAINBOW trial, and oxaliplatin is now frequently used as the first-line treatment for gastric cancer. Consequently, we think that a greater proportion of patients have residual neuropathy at the start of second-line treatment. In present trial, seven patients (20%) experienced a worsening of their grade during the study treatment. Three patients progressed from grade 0 to 1, two patients progressed from grade 0 to 2, and two patients progressed from grade 1 to 2. There were no changes in 25 patients, with twelve remaining at grade 0, eleven remaining at grade 1, and two remaining at grade 2. Three patients showed improvement from grade 1 to 0 during the trial treatment.

It is important to note that 10 out of 35 patients discontinued study treatment due to adverse events. All patients who discontinued treatment due to adverse events were male; however, there were no significant differences in baseline characteristics—such as age at treatment initiation, ECOG performance status, body weight, and body mass index—compared with those who did not discontinue due to adverse events. The adverse events leading to discontinuation were not attributable to a single toxicity; rather, in many cases, treatment was discontinued when multiple adverse events—such as fatigue, anorexia, mucositis, dysgeusia, and hypertension—occurred concurrently. This may reflect a characteristic of the present study, which evaluated second-line treatment for gastric cancer in a population with a relatively high median age. This treatment may require G-CSF prophylaxis and other aggressive supportive therapies. Effectively utilizing these supportive therapies allows one to fully realize the advantages of this treatment, such as managing with biweekly outpatient visits.

This study has several limitations. First, this is a single-arm phase II trial with a limited number of cases targeting only Japanese patients. Second, since the primary endpoint was ORR, patients with measurable lesions were eligible, and patients with only peritoneal dissemination or ascites were not included. There may be some bias in patients’ backgrounds. Several factors showed statistically significant differences in subgroup analyses for PFS and OS. However, the sample sizes were small, making it difficult to explain the reasons for these differences, and they may have been coincidental results specific to this trial. There was no significant difference in efficacy when the patient population was divided before and after the change permitting primary prophylactic administration of G-CSF. Third, information on supportive management for neuropathy was not collected. Further investigation is needed to determine whether supportive therapy can prevent the onset or worsening of neuropathy. Fourth, this trial did not conduct any biomarker studies, so the populations that would benefit most from this regimen were not identified.

In conclusion, the combination therapy of ramucirumab and docetaxel demonstrated some efficacy as a secondary treatment for AGC. However, caution is required regarding the occurrence of febrile neutropenia and other adverse events. Ramucirumab and docetaxel might be considered as an option for second-line treatment of patients with AGC.

## Data Availability

The datasets obtained from patients in this study may be used for future research purposes that are not specified at the time consent was obtained. In such cases, a new research protocol will be created or modified, and approval from the review committee and permission from the administrator of the implementing institution will be obtained. Furthermore, when providing data to other research institutions, it will be reported to the administrator of the implementing institution and provided only after anonymization.
